# Adoption of social networking sites among older adults: The role of the technology readiness and the generation to identifying segments

**DOI:** 10.1371/journal.pone.0284585

**Published:** 2023-04-18

**Authors:** Patricio E. Ramírez-Correa, Jorge Arenas-Gaitán, F. Javier Rondán-Cataluña, Elizabeth E. Grandon, Muriel Ramírez-Santana

**Affiliations:** 1 Engineering School, Universidad Católica del Norte, Coquimbo, Chile; 2 Department of Business Administration and Marketing, Universidad de Sevilla, Sevilla, España; 3 Department of Information Systems, Universidad del Bío-Bío, Concepción, Chile; 4 Public Health Department, Universidad Católica del Norte, Coquimbo, Chile; Fondazione Ugo Bordoni, ITALY

## Abstract

Older adults can take advantage of social networking sites (SNS). However, SNS are not without the access gap among elders. Assuming that the data are homogenous within the same population may not be precise in social science research. What is known about the heterogeneous nature of older people? Considering this issue and the lack of research to help reflect the heterogeneity of elderly users of technologies, this study aims to identify segments in the use of SNS by the elderly. Data were collected from older Chilean adults. Cluster analysis suggested different profiles of adult users regarding the Technology Readiness Index. We used a hybrid multigroup partial least squares-structural equation model, including the Pathmox algorithm, to identify segments in the structural model. Based on the technology readiness profiles and the generation, we identified three segments with different determinant effects to explain the intention to use SNS: independent elder, technological-apathetic elder, and technological-eager elder. The contributions from this study are triple. First, this study helps to better understand how the elderly adopt information technology. Second, this study complements the existing corpus of research on using the technology readiness index in the elderly population. Third, we used an innovative method to segment users in the acceptance technology model.

## Introduction

Understanding the factors that influence information technology adoption by older adults is crucial [1]. However, assuming homogeneity in a population can lead to inaccuracies in research findings. To ensure validity and robustness, hidden unobserved heterogeneity in samples of information technology users must be considered [2]. Although age, education, and prior technology experiences have been linked to information technology usage [3], other variables may affect behavioral differences, particularly among older adults. Despite the potential benefits [4, 5], there is a significant gap in information technology adoption among older adults [6, 7], highlighting the need to study these factors and promote adoption by all population segments. In Latin America, for instance, younger people (72%) are more likely to use the Internet than older adults (18%), regardless of the device used [8].

SNS can benefit older people by improving their independence, reducing isolation, and increasing social participation [7, 9, 10]. However, access gaps and the digital divide still exist among elders in all types of SNS [7, 9]. This study aims to identify segments in the use of SNS by the elderly, considering these issues and the lack of research that reflects the heterogeneity of elderly users of technology. Specifically, this study contributes in three ways: understanding how the elderly adopt information technology, complementing existing research on using the technology readiness index (TRI) in the elderly population, and using an innovative method to segment users in the acceptance technology model, employing a hybrid multigroup approach using Pathmox analysis to segment SNS elderly users.

### SNS and the elderly

SNS have attracted expert attention to foster social relationships among people, including older people [11]. In fact, in light of the framework of the ten priorities for older people to fit into the 2030 Agenda for Sustainable Development set by the World Health Organization [12], SNS appear as a crucial way to promote active aging. SNS can be used to reach health services, such as telemedicine, to connect medical doctors with older adults [13]. This fact is even more relevant in post-pandemic times. Although research on the use of SNS by the elderly is growing [14], the literature reported so far is concerning because very few data and specific studies are yet available on SNS use by the elderly [15].

Even though SNS have existed for almost two decades, a significant digital divide still affects the elderly [9]. Older people generally have lower information technology penetration rates than the rest [16]. In the case of digital technology, younger people see the Internet and SNS as a new virtual world where they can enjoy and express themselves and grow. This virtual environment is not recreational for older people but a simple communication tool [17]. Other reasons make older people less interested. They may not need to use these information technologies on a day-to-day basis. As a result, they lack experience using the Internet and SNS, which are associated with less need to seek information than other older groups [16]. All these elements militate in favor of the persistence of a stereotype of the elderly as clumsy and far from information technology [18]. However, this stereotyped image hides a richer reality. Learning about user segmentation is essential to guide public-policymakers in creating differentiated strategies to facilitate the transition toward technology use, particularly SNS, of elders according to the specific segments they belong to.

### Segmentation in SNS

Studies that assume data are homogeneous and representative of a single population may not be realistic in social sciences [19]. Since human behavior is usually not uniform and, therefore, a sample conceals different behaviors of the individuals that form it [20], it is appropriate to assume that there is a specific heterogeneity in measurable behaviors [21]. Concerning the heterogeneity of behavior, SNS use is not an exception. Since older people may be one of the most heterogeneous behavioral segments [22], the evolution of studies on heterogeneity in older people challenges the current validity of the technological stereotype associated with the elderly [14, 23]. Based on previous studies [24], we believe that the Technology Readiness Index and generation might explain segmentation in older users of SNS.

### Technology Readiness Index (TRI)

To better comprehend the adoption of social networking sites among older adults, it is vital to understand their technology readiness. The TRI, developed by Parasuraman [25], is a useful tool for assessing people’s willingness to use technology. The TRI represents a set of motivators and inhibitors influencing an individual’s predisposition to adopt cutting-edge technologies. This measure has been extensively used to assess technology predisposition in various cultures and for different types of technology. For instance, it has been used to measure the adoption of mobile payment applications among Indonesian users [26], attitudes towards emerging technologies such as autonomous cars, telemedicine, and enhanced living environments in Hungary [27], as well as the adoption of e-learning [28] and cutting-edge technologies among middle and high school students in India [29]. Moreover, TRI has been utilized to profile technology users globally. For instance, five profiles were identified in the USA: explorers, pioneers, skeptics, hesitators, and avoiders; three profiles in South Korea: explorers, laggards, and pioneers [30] profiles in South Africa; three of which shared similarities with the profiles of pioneers, paranoids, and explorers [31]; and five types of technology users in Chile: pioneer, hesitator, avoider, explorer, and skeptic [32].

In the context of older adults, TRI has been employed to examine the influence of personal beliefs on elderly individuals’ attitudes toward using online public services in Japan and the UK [33]. More recently, it was used to investigate the propensity to adopt and use technology for personal use among older adults in Finland [34]. The latter study found similar groups within working-age populations and, surprisingly, different groups than previous research with a mature target group. The pioneers belonged to the largest cluster, while skeptics and avoiders to the smallest clusters. The authors suggested that traditional beliefs about age and technology use are becoming outdated as technology becomes more widely adopted.

## Materials and methods

### Research model building

Two models stand out in explaining information technology usage: The Unified Theory of Acceptance and Use of Technology (UTAUT) and the Unified Theory of Acceptance and Use of Technology version 2 (UTAUT2). UTAUT was defined in response to the application of many ad hoc models that combine concepts from different theories to explain the phenomenon of acceptance of new technology. Venkatesh *et al*. [35] proposed the need for synthesis to progress toward a unified vision of information technology (IT) acceptance. UTAUT highlights four key variables as direct drivers of the intention to use the technology: effort expectancy (EE), performance expectancy (PE), social influence (SI), and facilitating conditions (FC). In turn, the intention and FC explain the use of the technologies. Founded on UTAUT, Venkatesh *et al*. [36] created a new model coined UTAUT2 to be applied to a broader context, incorporating three new determinants of intention: hedonic motivation (HM), price-value (PV), and habit (HA). In addition, this last variable is directly associated with the use of the technologies.

Considering the non-homogeneous behavior of SNS users, we propose exploring the non-observed heterogeneity in the research model by examining significant segmentation in older adults’ perceptions of their adoption of SNS. In addition to the control variables proposed by UTAUT2 (age, gender, and experience), psychographic variables will be analyzed, such as educational level, technology readiness, income, employment status, and generation. Therefore, our proposal is as follows:

**Proposition:** There are significantly different segments of Chilean older adults who adopt SNS.

**Fig 1:**
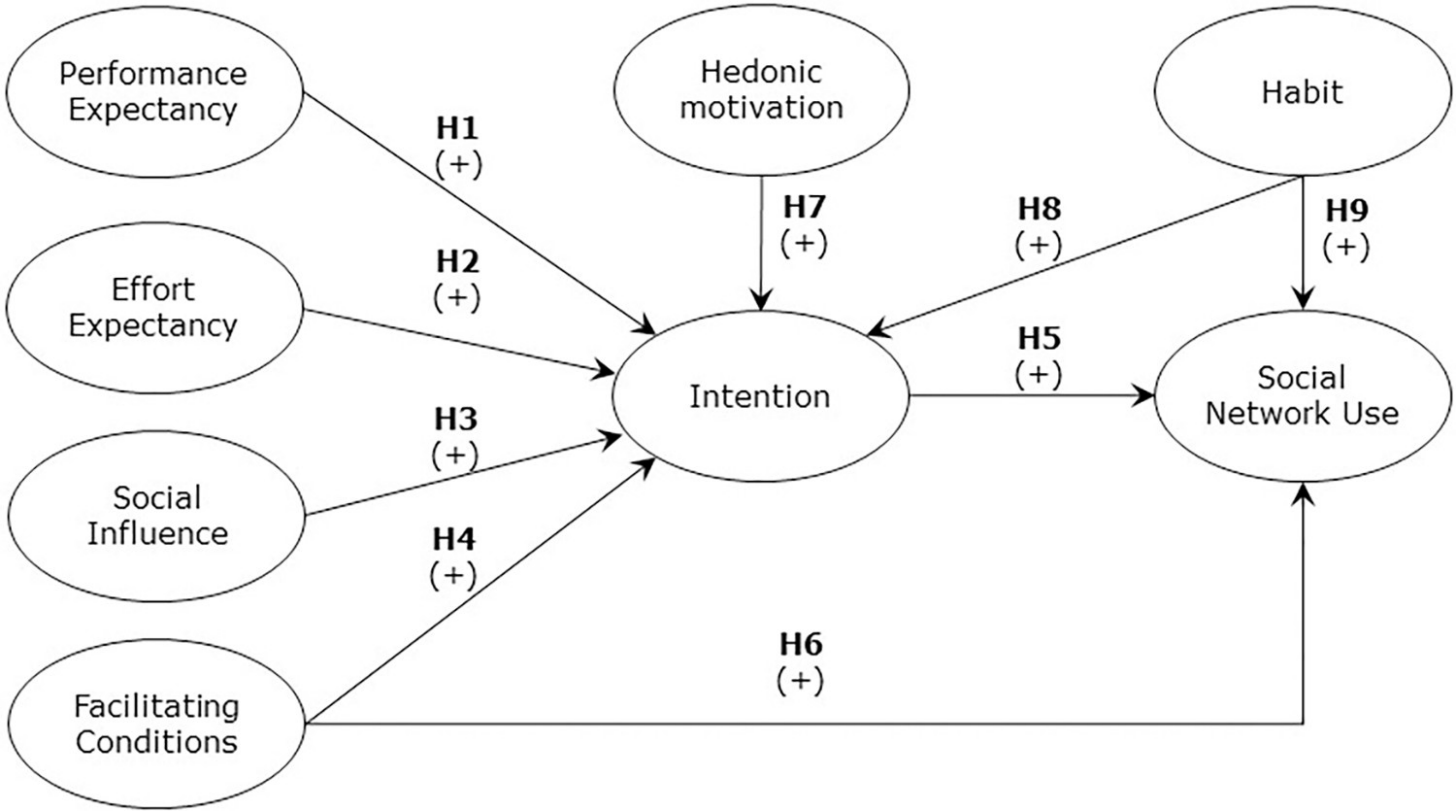
Research model and hypotheses.

Given that previous studies have successfully applied UTAUT2 to explain the acceptance of IT in Chile [37] and IT by older adults in Spain [38] and Portugal [39], this theoretical structure has been taken as the basis for this research. Fig 1 illustrates the research model as well as the hypotheses.

Behavioral intention is the way an individual has formulated pre-arranged plans for whether to perform a specific future behavior [40]. Several studies propose variables that explain the rise in the intention to use or continue to use SNS. On the one hand, the literature shows that PE, the extent to which the use of technology will provide benefits to individuals in the conduct of specific activities[35], impacts the intention to use SNS [41, 42]. It is also indicated that EE, the degree of ease associated with the use of technology by people [35], impacts the behavioral intention to use SNS [41, 42]. Moreover, some studies propose that SI, the extent to which people perceive that individuals who are important to them believe that they should use a particular technology [40], impacts the behavioral intention to use SNS [43, 44]. Finally, studies suggest that FC, the perception that resources and support are available to execute an action [35], accounts for the increase in behavioral intention of using information technology [36, 37], and in particular, the intention to use SNS for purchases [45].

Based on the previous studies, we proposed the following hypotheses:

**H1:** PE is positively associated with the intention to use SNS in older Chilean adults.

**H2:** EE is positively associated with the intention to use SNS in older Chilean adults.

**H3:** SI is positively associated with the intention to use SNS in older Chilean adults.

**H4:** FC are positively associated with using SNS in older Chilean adults.

Usage behavior is defined as the frequency of use IT (Davis, 1989) and has been used as the last consequence in all IT acceptance models. The literature suggests that behavioral intention explains SNS use [46–48]. On the other hand, it is indicated that FC influence the use of SNS [45, 49–52]. Additionally, the intention to use SNS could be predicted by two variables associated with the context of user consumption. First, HM, defined as the pleasure derived from technology, has been proposed as an antecedent to the intention to use SNS [45, 52, 53]. Second, HA, defined as the extent to which people tend to engage in automated behaviors due to learning, has been considered a determinant of the intention and the use of SNS [45, 52, 53]Click or tap here to enter text. Based on the foregoing, the following hypotheses have been proposed:

**H5:** The intention to use SNS is positively associated with SNS use in older Chilean adults.

**H6:** FC are positively associated with SNS use in older Chilean adults.

**H7:** HM is positively associated with the intention to use SNS in older Chilean adults.

**H8:** HA is positively associated with the intention to use SNS in older Chilean adults.

**H9:** HA is positively associated with SNS use in older Chilean adults.

### Methodology

**Type of study.** Descriptive, correlational, and cross-sectional.

#### Questionnaire

The questionnaire was composed of 52 questions. Original scales belong to UTAUT models and all of them have been adapted from previous studies related to SNS use (see S1 File). The original scales were made in English [36, 54], and further studies have validated these scales translated into Spanish for general or elderly users of information technology [37, 55]. We measured the constructs using a 5-point Likert scale that ranged from "strongly disagree" to "strongly agree." Besides, 16 items associated with TRI were included [32, 56]. A pretest of items from the TRI was conducted to evaluate their adequacy and quality before including them in the final questionnaire. We also measured age, gender, educational level, work and retirement status, socioeconomic class, and Internet experience.

#### Data collection

Data from elderly Internet users were collected through face-to-face interviews based on the questionnaire. A user was considered a person who reports using the Internet for three months or less, regardless of whether they are an SNS user or non-user. The inclusion criteria for a subject in the sample were adults 60 years old or more, self-reliant, and Internet users.

Due to the nature of the study, we stratified the sample. The regions of Coquimbo and Biobío are considered since they have a higher proportion of the population over 60 in Chile. Since the previous literature has indicated the importance of classifying people by generation to explain their Internet behavior in Chile [24], we used generation as a variable for stratification. In Chile, the population over 60 includes two generations: silent and baby boomers. In agreement with the previous literature, we distinguished two segments in the Chilean baby boomer generation: early and late baby boomers. Early baby boomers included Chileans born between 1947 and 1955, and late baby boomers included Chileans born between 1956 and 1961.

Additionally, we thought about the gender variable because, among older people, society assumes different objectives and has raised men and women differently [57]. In determining the proportion of Internet users by generation and gender, we use Chile’s latest official survey on Internet access and use. Once this database was filtered and ratios calculated, 162,639 older adults were identified. A single apposition stratified method was proposed to determine the sample size, considering a maximum error of 5%. The overall sample was calculated as 383. The overall sample is spread across the strata by simple affixing. The stratified sample distribution shows 124 from Coquimbo (51 late baby boomers, 41 early baby boomers, and 32 silent generation) and 259 from Biobío (109 late baby boomers, 87 early baby boomers, and 63 silent generation). The sample consists of 170 males, 213 females, 160 of the late baby boomers generation, 128 of the early baby boom generation, and 95 members of the silent generation. Six surveyors collected the data for four weeks in September 2021.

The study was undertaken following the Declaration of Helsinki and endorsed by the Local Ethics Committee of the Universidad Católica del Norte (R05/2021).

#### Data analysis

**Fig 2:**
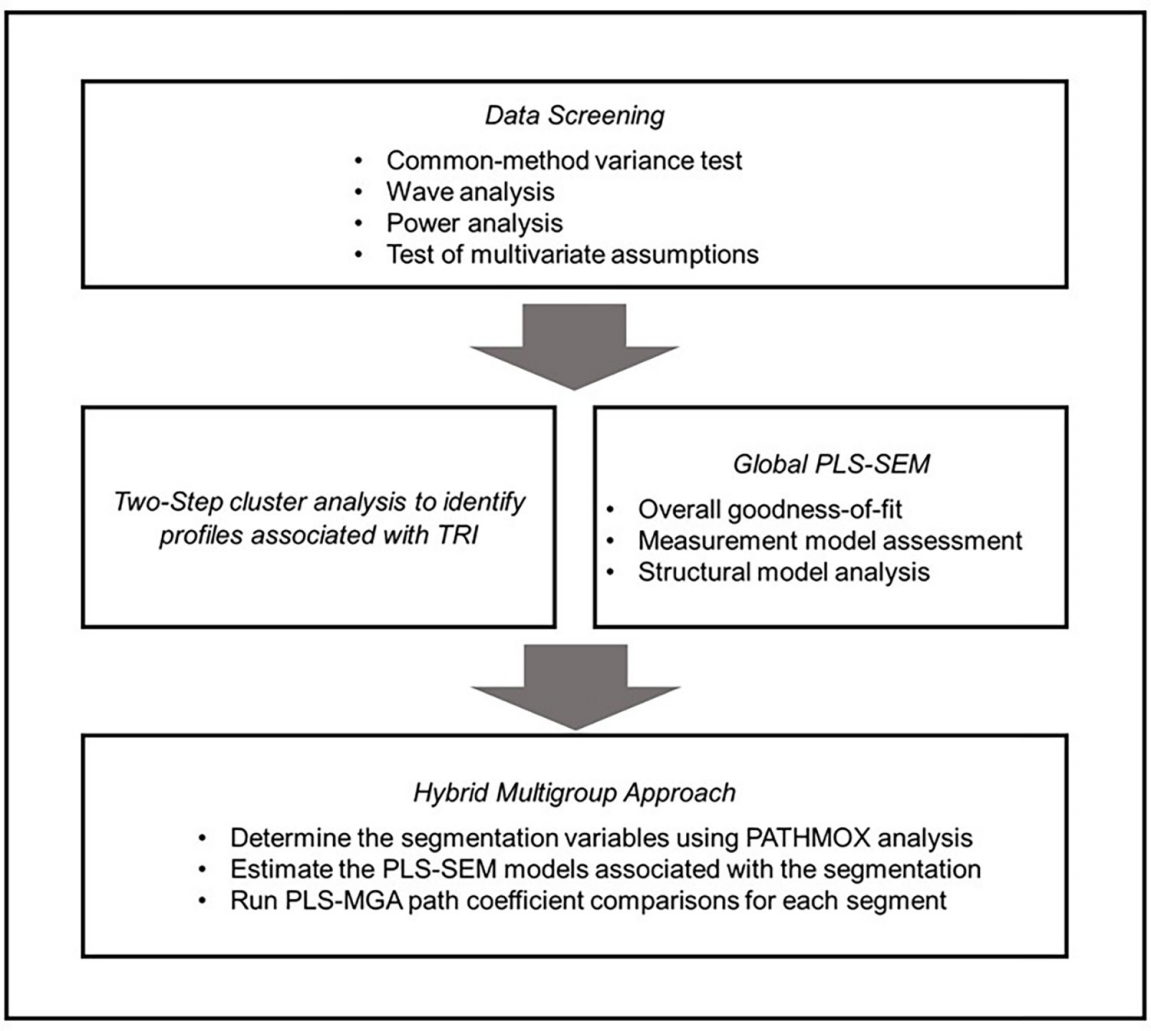
The general process of data analysis.

Fig 2 shows the general process of data analysis.

*Data screening*. The primary data were screened for common method bias (CMB) using Harman’s single-factor test [58] and Kock’s method [59]. The results showed that there was no CMB present in the study data. A wave analysis was also conducted to ensure no bias due to non-response, and the results indicated no significant differences between early and late respondents. The required sample size was determined using the gamma-exponential method [60], and it was found that the study’s total sample size met the minimum requirement. Four multivariate assumptions were tested using the Kolmogorov-Smirnov test, residual normalized regression scatter graph, VIF, and linearity deviation tests. Nonlinear relationships were found between the intention to use SNS with PE and EE, but there were no concerns regarding non-normal distribution, homoscedasticity, multicollinearity, or linearity deviation.

#### Analysis of unobserved heterogeneity through the modeling of latent classes with Pathmox

PLS-SEM has traditionally assumed that the analyzed data originates from a homogeneous population. However, this assumption is often unrealistic in social sciences and the study of human behavior because individuals may have varying perceptions and evaluations of a phenomenon [61, 62]. This heterogeneity within a population can impact both the measurement and structural models of a PLS-SEM analysis. Although identifying the sources of heterogeneity in the data can be complex, failing to account for it when there are significant differences among the population segments can result in biased PLS-SEM results [63]. To account for population heterogeneity in PLS-SEM analysis, the population can be divided into homogeneous segments using a priori variables or clustering methods. However, these methods have limitations, such as needing a substantive theory [62, 64] or restrictive statistical models [65].

Pathmox is a new algorithm for PLS-SEM segmentation that generates a tree with various models in each node, using binary segmentation to detect heterogeneity in PLS-SEM analysis. It requires three parameters: significance threshold, minimum individuals per node, and the number of levels allowed to grow. In addition, the algorithm uses an F-test to compare parent and child node path coefficients to determine optimal partitions and binary splits for the models [66]. A drawback of Pathmox is that it can only identify the most significant models that differ in terms of available sources of heterogeneity. To address this limitation, a hybrid multigroup approach in three steps was proposed [66]: determine segmentation variables, estimate PLS-SEM models, and run PLS-MGA path coefficient comparisons for each segment.

*Cluster analysis*. A two-step cluster analysis was used to identify the different profiles associated with TRI. Two-step cluster analysis is a statistical technique used to group similar cases or observations in a dataset containing continuous and categorical variables. This technique involves a two-step process, starting with a pre-clustering step that divides the dataset into small sub-clusters using hierarchical clustering. These sub-clusters are then analyzed in the second step using a model-based clustering technique that uses a probabilistic model to identify the final clusters. Typically, a mixture model is used in this step, assuming that each cluster’s data comes from a specific distribution. This technique is broadly used in various fields to identify sub-groups of individuals or cases with similar characteristics.

*Software*. The SPSS 27 software was used for clustering, the SmartPLS 3.3.3 software provided the model estimates (global and multigroup analysis), and the genPathmox package in R software version 0.7 provided the Pathmox analysis.

## Results

### Technology readiness

Table 1 shows the results of the cluster analysis; this procedure identified five clusters. The clusters have been labeled based on the proposal of Parasuraman and Colby [56] and its recent application in a Chilean consumers sample of Ramírez-Correa *et al*. [32] as follows: Hesitators (Cluster 1), Explorers (Cluster 2), Skeptics (Cluster 3), Avoiders (Cluster 4), and Pioneers (Cluster 5).

**Table 1:** Cluster means and ranking.

Label	Cluster	Optimism	Innovativeness	Discomfort	Insecurity	N	%
Hesitators	Cluster 1	4.23 (3)	3.37 (2)	3.57 (3)	4.22 (2)	72	18.8%
Explorers	Cluster 2	4.48 (1)	3.67 (1)	2.65 (5)	3.01 (5)	101	26.4%
Skeptics	Cluster 3	2.85 (5)	2.58 (3)	3.23 (4)	3.17 (4)	43	11.2%
Avoiders	Cluster 4	3.43 (4)	1.86 (5)	4.05 (1)	4.40 (1)	96	25.1%
Pioneers	Cluster 5	4.25 (2)	2.25 (4)	3.58 (2)	3.71 (3)	71	18.5%

Hesitators (Cluster 1) correspond to 18.8% of the sample. In this cluster of cautious “Hesitators,” both motivators and inhibitors’ scores of adopting cutting-edge technology are relatively moderate. Explorers (Cluster 2) is the largest cluster and corresponds to 26.4% of the sample. In this cluster, the highly tech-oriented “explorers” show the highest score motivators and lowest score inhibitors of adopting cutting-edge technology. Skeptics (Cluster 3) is the smallest cluster and corresponds to 11.2% of the sample. The dispassionate “skeptics” show the lowest optimism and moderate innovativeness and, on the other hand, lower inhibitors of adopting cutting-edge technology. Avoiders (Cluster 4) correspond to 24.1% of the sample. The tech-resistant “avoiders” in this cluster show the lowest score motivators and highest score inhibitors of adopting cutting-edge technology. Pioneers (Cluster 5) represent 18.5% of the sample. In this cluster, the engaged “pioneers” show a high score in optimism and discomfort and moderate scores in innovativeness and insecurity. The scores of this cluster change a little from those of the profiles in other studies with a general sample [32] but close to studies with an elderly sample [34].

### Global PLS‑SEM

The overall goodness-of-fit of the model fit is the starting point for the PLS-SEM assessment because if the model does not match the data, the data contains more information than the model conveys. We used fit indices to examine the overall goodness-of-fit of the global model. Table 2 presents the result. The assessment of the standardized root means square residual (SRMR) index offers a good value of 0.063, below the threshold value of 0.08 proposed by Hu and Bentler [67]. Given these indices, the model fits well.

**Table 2:** Goodness of model fit.

Fit criteria	Saturated model	Estimated model
SRMR	0.061	0.063
d_ULS_	1.514	1.608
d_G_	0.725	0.734

**Notes:** Standardized Root Mean Square Residual (SRMR)

Unweighted Least Squares Discrepancy (d_ULS_), Geodesic Discrepancy (d_G_).

Before the structural analysis, the reliability and validity of the measurement model were examined. Except for the variable use of social networks modeled as a composite construct, all the latent variables were modeled with a reflexive approach. Each variable is an antecedent of its indicators in a reflexive construct. In a composite construct, indicators do not provoke and do not reflect the variable but make it up [68]. As presented in Table 3, reliability was assessed by observing individual loads or simple correlations between measurements and their corresponding construct. All the loads exceed the minimum value of 0.5 and most often exceed the value of 0.8. For U the weights are shown in italics.

**Table 3:** Discriminant validity—Cross loadings (loadings and weights).

Item	FC	EE	PE	HA	IU	HM	SI	U
FC1	**0.778**	0.478	0.398	0.481	0.361	0.467	0.272	0.339
FC2	**0.805**	0.741	0.388	0.547	0.393	0.435	0.278	0.477
FC3	**0.808**	0.381	0.501	0.431	0.491	0.465	0.445	0.456
EE1	0.649	**0.931**	0.495	0.598	0.441	0.478	0.314	0.541
EE2	0.621	**0.953**	0.470	0.560	0.366	0.441	0.247	0.473
EE3	0.594	**0.923**	0.406	0.511	0.336	0.433	0.238	0.443
PE1	0.412	0.436	**0.763**	0.489	0.456	0.504	0.411	0.532
PE2	0.471	0.450	**0.870**	0.499	0.596	0.556	0.457	0.567
PE3	0.417	0.333	**0.832**	0.382	0.510	0.536	0.492	0.398
PE4	0.465	0.388	**0.789**	0.429	0.542	0.493	0.468	0.423
HA1	0.552	0.573	0.543	**0.896**	0.573	0.628	0.353	0.692
HA2	0.524	0.546	0.528	**0.935**	0.557	0.570	0.402	0.702
HA3	0.645	0.600	0.441	**0.854**	0.567	0.599	0.376	0.620
HA4	0.505	0.492	0.485	**0.904**	0.571	0.560	0.450	0.687
HA5	0.518	0.491	0.483	**0.913**	0.568	0.526	0.423	0.709
IU1	0.524	0.377	0.603	0.580	**0.915**	0.566	0.511	0.571
IU2	0.433	0.374	0.580	0.566	**0.906**	0.555	0.528	0.530
HM1	0.543	0.455	0.624	0.576	0.579	**0.919**	0.404	0.550
HM2	0.521	0.468	0.565	0.595	0.545	**0.912**	0.435	0.503
HM3	0.509	0.415	0.579	0.595	0.574	**0.930**	0.404	0.546
SI1	0.343	0.235	0.442	0.328	0.450	0.360	**0.860**	0.363
SI2	0.332	0.201	0.459	0.329	0.430	0.357	**0.884**	0.376
SI3	0.360	0.180	0.480	0.400	0.477	0.401	**0.857**	0.382
SI4	0.382	0.335	0.493	0.422	0.539	0.385	**0.767**	0.427
U1	0.333	0.411	0.422	0.584	0.483	0.431	0.342	*0*.*340*
U2	0.423	0.373	0.447	0.528	0.522	0.487	0.346	*0*.*256*
U3	0.475	0.422	0.529	0.666	0.482	0.499	0.398	*0*.*444*
U4	0.440	0.418	0.393	0.529	0.381	0.357	0.339	*0*.*242*

**Notes**: Bold values are loadings for each item that is above the recommended value of 0.5. Also, an item’s loadings on its own variable are higher than all of its cross-loadings with other variables. For U the weights are shown in italics.

Facilitating conditions (FC), effort expectancy (EE), performance expectancy (PE), habit (HA), intention to use (IU), hedonic motivation (HM), social influence (SI), and use (U).

We used Cronbach’s alpha (CA) coefficient as the reliability index of the reflective constructs. Moreover, we calculated the composite reliability (CR) for each of these constructs. The values of this index range from 0.839 to 0.956. The Average Variance Extracted (AVE) was calculated for each reflective construct to evaluate convergent validity. Given that the values are above the required minimum level of 0.5, it can be concluded that all these constructions have convergent validity. Table 4 exhibits the construct coefficients.

**Table 4:** Construct coefficients.

Construct	CA	Rho A	CR	AVE
FC	0.716	0.724	0.839	0.635
EE	0.930	0.947	0.955	0.876
PE	0.831	0.840	0.887	0.664
HA	0.942	0.943	0.956	0.812
IU	0.794	0.795	0.906	0.829
HM	0.910	0.911	0.943	0.847
SI	0.864	0.864	0.907	0.711
U	Composite			

**Notes:** Cronbach’s alpha (CA), composite reliability (CR), average variance extracted (AVE), facilitating conditions (FC), effort expectancy (EE), performance expectancy (PE), habit (HA), intention to use (IU), hedonic motivation (HM), social influence (SI), and use (U).

We employed two tests to evaluate the discriminant validity of the constructs: the Fornell-Larcker test by analyzing if the square root of AVE of each construct is greater than the correlations with the rest of the constructs (see Table 5). Second, the Heterotrait-Monotrait (HTMT) ratio reveals scores under the threshold of 0.9 (see Table 6). These results demonstrate the discriminant validity.

**Table 5:** Construct discriminant validity—Fornell-Larcker criterion.

Construct	FC	EE	PE	HA	IU	HM	SI	U
FC	0.797							
EE	0.667	0.936						
PE	0.543	0.493	0.815					
HA	0.607	0.599	0.551	0.901				
IU	0.527	0.413	0.650	0.630	0.910			
HM	0.570	0.484	0.641	0.639	0.616	0.920		
SI	0.425	0.289	0.561	0.445	0.570	0.450	0.843	
U	0.540	0.524	0.589	0.758	0.605	0.580	0.464	Composite

**Notes:** Facilitating conditions (FC), effort expectancy (EE), performance expectancy (PE), habit (HA), intention to use (IU), hedonic motivation (HM), social influence (SI), and use (U).

**Table 6:** Construct discriminant validity—Heterotrait-Monotrait Ratio.

Construct	FC	EE	PE	HA	IU	HM
EE	0.815					
PE	0.695	0.556				
HA	0.744	0.636	0.624			
IU	0.688	0.474	0.795	0.728		
HM	0.707	0.524	0.737	0.692	0.724	
SI	0.523	0.310	0.657	0.486	0.680	0.504

**Notes:** Facilitating conditions (FC), effort expectancy (EE), performance expectancy (PE), habit (HA), intention to use (IU), hedonic motivation (HM), and social influence (SI).

We evaluated the composite construct’s external validity based on the saturated model’s overall model fit tests (Table 2). We assume that the indicators form the composite as per the proposed measurement model given the results. Furthermore, the composite construct estimated in Mode B has been evaluated according to discriminant validity, potential multicollinearity, and magnitude of weights. Discriminant validity is reached where correlations between components and other latent variables are less than 0.7. As shown in Table 7, the highest Kendall Tau-b correlation coefficient is 0.574, indicating that discriminant validity is reached. Furthermore, as shown in Table 8, the maximum value of the variance inflation factor (VIF) is 1.708, which is lower than the threshold of 3.3, meaning that there is no multicollinearity problem among the indicators for each latent variable. In addition, the sign, magnitude, and statistical significance of the weights were evaluated. Based on this review, it is argued that the indicators make an essential contribution to its composite.

**Table 7:** Correlations.

Construct	FC	EE	PE	HA	IU	HM	SI	U
FC	1	0.505	0.473	0.472	0.462	0.495	0.389	0.389
EE	0.505	1	0.364	0.460	0.317	0.378	0.222	0.383
PE	0.473	0.364	1	0.422	0.527	0.508	0.470	0.448
HA	0.472	0.460	0.422	1	0.512	0.521	0.327	0.574
IU	0.462	0.317	0.527	0.512	1	0.524	0.488	0.463
HM	0.495	0.378	0.508	0.521	0.524	1	0.367	0.438
SI	0.389	0.222	0.470	0.327	0.488	0.367	1	0.345
U	0.389	0.383	0.448	0.574	0.463	0.438	0.345	1

**Notes:** Facilitating conditions (FC), effort expectancy (EE), performance expectancy (PE), habit (HA), intention to use (IU), hedonic motivation (HM), social influence (SI), and use (U).

**Table 8:** VIF and weight of indicators of the composite construct.

Indicator	VIF	Weight
U1	1.459	0.340***
U2	1.501	0.256***
U3	1.708	0.444***
U4	1.422	0.242***

**Notes:** *** significant p < 0.001(2-tailed), VIF: Variance inflation factor.

**Fig 3:**
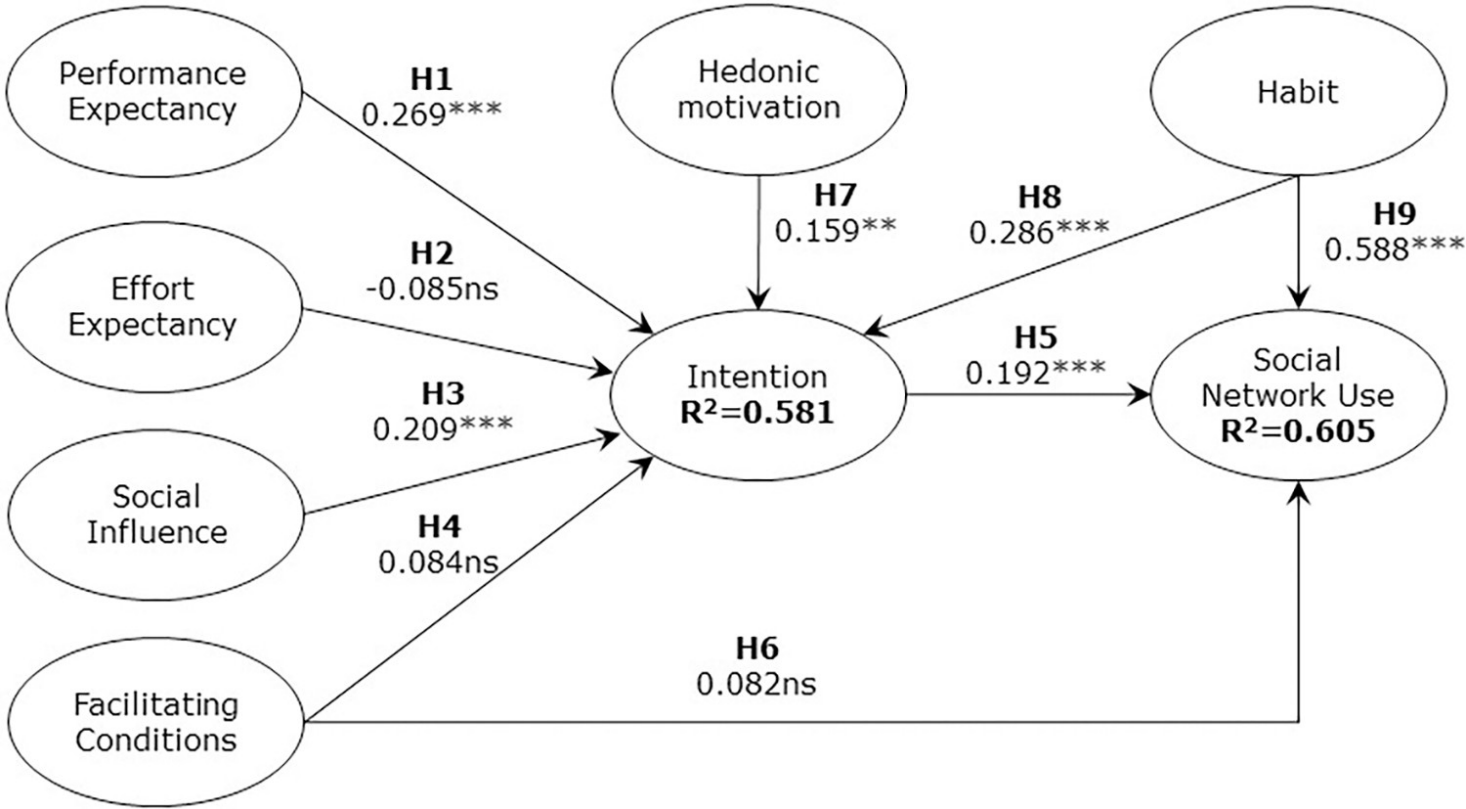
PLS results of the global model.

Following an assessment of the measurement model, the analysis of relationships among the latent variables of the model was examined. As can be seen in Table 9, the hypotheses were verified by examining the path coefficients. We computed a bootstrapping of 5,000 sub-samples to demonstrate the statistical significance of each path. The variance explained (R^2^) of endogenous variables served as an indicator of the explanatory power of the global model. Fig 3 illustrates the PLS-SEM results of the global model. Along with the explained variance of the global model, we calculated the Q^2^ of the endogenous variables. Q^2^ measures the extent to which observed values are reproduced by the model and its parameter estimates through cross-validation. The findings show the predictive relevance of the global model.

**Table 9:** Path coefficients of global model.

H.	Path	Beta	T statistics	P values	2.5%	97.5%
H1	PE ->IU	**0.269**	4.553	0.000	0.153	0.383
H2	EE ->IU	-0.085	1.739	0.082	-0.178	0.012
H3	SI ->IU	**0.209**	4.221	0.000	0.113	0.308
H4	FC->IU	0.084	1.542	0.123	-0.017	0.192
H5	IU-> U	**0.192**	4.058	0.000	0.102	0.286
H6	FC->U	0.082	1.715	0.086	-0.009	0.174
H7	HM->IU	**0.159**	3.215	0.001	0.059	0.254
H8	HA->IU	**0.286**	5.659	0.000	0.188	0.386
H9	HA->U	**0.588**	12.192	0.000	0.492	0.680
		R^2^	R^2^ adj.	Q^2^		
IU		0.581	0.575	0.466		
U		0.605	0.602	0.348		

**Notes:** Facilitating conditions (FC), effort expectancy (EE), performance expectancy (PE), habit (HA), intention to use (IU), hedonic motivation (HM), social influence (SI), and use (U).

### Hybrid multigroup PLS‑SEM results

#### Step 1: Determining the hybrid segmentation variable

Pathmox analysis was conducted using the following list of variables: generation (silent, early baby boomers, and late baby boomers), gender (male and female), experience with Internet (low, moderate, and high), educational level (no education, primary, secondary, and tertiary), socio-economical class (lower, lower-middle, middle, upper-middle, and upper), working status (non-working and working), retired status (non-retired and retired), and profiles associated with the technology readiness index (explorer, pioneer, skeptic, hesitator, and avoider).

**Fig 4:**
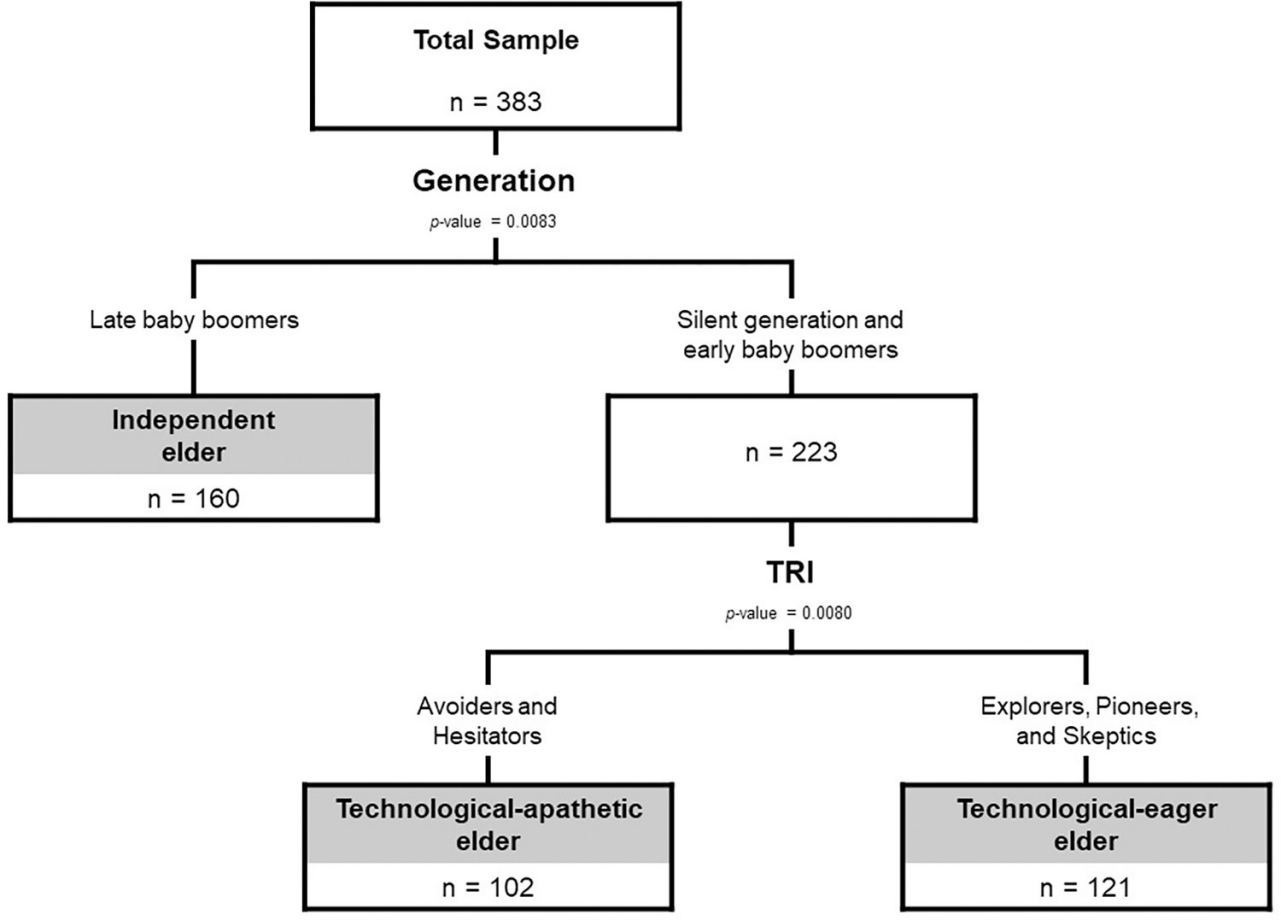
Pathmox tree.

An optimized procedure based on the mean of R^2^ was included to identify the minimum permissible size for a node and the maximum depth of levels, the former ranging from 0.2 to 0.5 and the latter ranging from 2 to 10, while the threshold significance for the partitioning algorithm was p = 0.05. The resultant Pathmox tree shown in Fig 4 consisted of three-terminal nodes.

Pathmox chose generation as a variable with the greatest power to distinguish older adults who use social networks. According to their TRI-related profile, older adults in the silent generation and early baby boomers were more differentiated. As a result, we identified three segments: the independent elder, the technological-apathetic elder, and the technological-eager elder. These three segments are subsequently used in the PLS-MGA to compare the path coefficients.

#### Step 2: Estimate the PLS-SEM models associated with the previously determined segmentation

Table 10 shows the path coefficients, R^2^ and Q^2^, for each segment (details of analyses by segments in S2 File). In the “independent elder” segment, PE and HA explained the intention to use SNS. In the segment labeled “technological-apathetic elder,” PE, HA, and SI explained the intention of use. Finally, for the users in the segment marked as “technological-eager elder,” SI, HM, and HA explained the intention of use. HA is important in all three segments to explain the intention to use but at different levels. On the other hand, PE is important in the first two segments, and SI is important in the latter two segments to explain this intention.

**Table 10:** Path coefficients for each group.

Path	Independent elder (N = 160)			Technological-apathetic elder (N = 102)			Technological-eager elder (N = 121)		
	Beta	T stat.	p-value	Beta	T stat.	p-value	Beta	T stat.	p-value
PE ->IU	**0.232**	2.962	0.003	**0.410**	3.777	0.000	0.096	0.961	0.337
EE ->IU	-0.057	0.761	0.447	-0.099	0.942	0.346	-0.042	0.608	0.543
SI ->IU	0.075	1.117	0.264	**0.211**	2.543	0.011	**0.421**	3.774	0.000
FC->IU	0.135	1.659	0.097	0.027	0.274	0.784	0.119	1.395	0.163
IU-> U	**0.370**	4.692	0.000	-0.026	0.239	0.811	**0.250**	3.240	0.001
FC->U	0.038	0.409	0.683	0.066	0.670	0.503	0.068	0.884	0.377
HM->IU	0.125	1.463	0.144	0.137	1.508	0.132	**0.175**	2.080	0.038
HA->IU	**0.413**	4.681	0.000	**0.251**	3.138	0.002	**0.166**	1.962	0.050
HA->U	**0.462**	5.179	0.000	**0.693**	7.471	0.000	**0.583**	8.662	0.000
	R^2^	R^2^ adj.	Q^2^	R^2^	R^2^ adj.	Q^2^	R^2^	R^2^ adj.	Q^2^
IU	0.598	0.582	0.464	0.604	0.579	0.467	0.599	0.578	0.456
U	0.627	0.620	0.375	0.517	0.503	0.213	0.633	0.623	0.338

**Notes:** Facilitating conditions (FC), effort expectancy (EE), performance expectancy (PE), habit (HA), intention to use (IU), hedonic motivation (HM), social influence (SI), and use (U).

#### Step 3: Run PLS-MGA path coefficient comparisons for each segment

To guarantee that differences in model estimation do not arise from distinct content or meaning for constructs within groups, before conducting the group comparison in PLS-SEM, we verified the measurement invariance using the Measurement Invariance of Composite Models (MICOM) procedure [69]. First, we established the configural invariance; each construct has been specified the same for all the groups. Next, we verified the compositional invariance; composite scores measure the same construct across groups. Compositional invariance was confirmed in all cases since the original correlations are equal to or larger than the 5% quantile [69]. For instance, for the FC construct, the score correlation between the independent and technological-apathetic elder groups is 0.996, which is greater than the 5% quantile of 0.989. For the same construct, the score correlation between the independent and technological-eager elder groups is 0.996, which is greater than the 5% quantile of 0.990. Finally, when comparing the technological-apathetic elder with the technological-eager elder groups, the results indicate a score correlation of 0.984 for the FC construct, which is greater than the 5% quantile of 0.983. The results show the same pattern for each construct when comparing between groups. These results allowed us to proceed with the PLS-MGA. Table 11 shows the PLS-MGA results for the model with all three segments. PLS-MGA allows us to verify if the predefined datasets have significant differences in their estimates of group-specific parameters. In particular, we examined significant path coefficient differences among the previously identified three groups. PLS-MGA compares group pairs by following these steps. First, the number of observations in two comparison groups must be known; second, the path coefficients of each group to be compared are estimated individually; third, standard errors in parameter estimates for each group are determined through bootstrapping; and lastly, a statistical test offered by Keil *et al*. [70] is computed.

**Table 11:** Path coefficients’ differences and significance levels.

Path	Independent elder	Technological-apathetic elder	Technological-eager elder	Independent elder versus technological-apathetic elder	Independent elder versus technological-eager elder	Technological -apathetic elder versus technological-eager elder
PE ->IU	0.232	0.410	0.096	-0.178	0.136	**0.313** *
EE ->IU	-0.057	-0.099	-0.042	0.042	-0.015	-0.057
SI ->IU	0.075	0.211	0.421	-0.136	**-0.346** **	-0.210
FC->IU	0.135	0.027	0.119	0.109	0.016	-0.092
IU-> U	0.370	-0.026	0.250	0.397***	0.120	**-0.277** *
FC->U	0.038	0.066	0.068	-0.028	-0.030	-0.002
HM->IU	0.125	0.137	0.175	-0.013	-0.051	-0.038
HA->IU	0.413	0.251	0.166	0.163	**0.247** *	0.084
HA->U	0.462	0.693	0.583	-0.231	-0.121	0.110

**Notes:** Facilitating conditions (FC), effort expectancy (EE), performance expectancy (PE), habit (HA), intention to use (IU), hedonic motivation (HM), social influence (SI), and use (U).

* p-value <0.001

** p-value <0.01, * p-value <0.05.

We found that for explaining intention to use, the PE was significantly more important for a technological-apathetic elder than for a technological-eager elder (p-value <0.05), that the SI was significantly less important for an independent elder than for a technological-eager elder (p-value <0.01). Lastly, we found that the HA was significantly more important for an independent elder than for a technological-eager elder (p-value <0.05). Additionally, for explaining the use, intention to use was significantly more important for an independent elder than for a technological-apathetic elder (p-value <0.001) and was significantly less important for a technological-apathetic elder than for a technological-eager elder (p-value <0.05).

## Discussion

Although the COVID-19 pandemic has led to a massive and immediate change toward online platforms, not participating in the digital world would create a dual sense of social exclusion in times of physical distancing for older adults. Due to COVID-19, seniors were notably excluded from face-to-face society. In addition, older adults are members of a population frequently excluded from digital services because they choose not to use new technologies. This missing digital engagement includes valuable online services and content, such as SNS [6]. However, studies declare that, at least for some older adults, this painful experience has increased their digital life. For these older adults, this process progressed in stages from total confusion to relative ease in using technology, reducing loneliness, and changing the way they lead their lives. This transit was a practical demonstration of the saying "there is no age to learn,” where the SNS provided an excellent way to speak and express oneself, a healing mechanism for the elderly population [71].

Notwithstanding the situation described above, technology adoption in older adults is not fully explained. While segmentation can improve this explanation, validating functional segmentation is a debt to society. A recent systematic literature review on elderly’s technology adoption identifies gender, age, voluntariness of use, experience, and purchase behavior as moderators in research models [1]. All these moderators are generic variables vastly used to improve the explanation of the adoption of technologies but do not arise specifically from the context of the older adult population. Our study proposes and validates two new specific variables to segment the adoption in this age group: generation and TRI profiles.

To implement our proposal, we use a recent and practical process incorporating data science elements, such as decision tree analysis, with multi-group analysis in structural equation models. Although this proposal is based on the work of Lamberti [66], we have added a parameter search activity in the decision tree generation process that improves the pursuit for a better explanation of the average variance in the detected segments. Additionally, we carefully frame the entire data analysis process so that it can be used in other studies.

Regarding elderly user profiles, the two-step cluster analysis yielded five groups: hesitators, explorers, skeptics, avoiders, and pioneers. These results are in line with Parasuraman and Colby’s technology users’ profiles [56] as well as Ramírez-Correa *et al*. [32] in a study conducted with consumers of different ages in Chile. The results are mainly congruent with Sell and Walden’s work [34] which, using two-step cluster analysis, found the same profiles in an older population in Finland. Explorers were the largest group in this study and the second largest in Sell and Walden’s research. The skeptic profile represented the smallest group in this study and the second smallest, almost the smallest in the study by Sell and Walden. The mean values of every cluster were similar throughout the four TRI dimensions in both studies.

The results support the research model’s hypotheses H1, H3, H5, H7, H8, and H9. The intention to use SNS is explained from greater to lesser importance by the variables HA, PE, SI, and HM. Similarly, the use of SNS is explained from greater to lesser importance by the variables HA and intention to use. These findings also support the proposition of the study: There are significantly different segments of Chilean older adults who adopt SNS. Specifically, the generation and the profile associated with TRI segment the acceptance of SNS into three groups: independent elders, technological-apathetic elders, and technological-eager elders. Furthermore, there are statistically significant differences in the effects of the variables that explain the intention to use SNS (PE, SI, and HA) and the use of SNS (intention to use) among these elderly groups.

Identifying distinct segments in adopting SNS among older adults in Chile highlights the need to acknowledge the diversity within this demographic group. Furthermore, this finding is consistent with previous research, demonstrating the significance of recognizing different segments when examining technology adoption among older adults [72, 73]. For instance, one study [72] identified three segments of older adults based on their attitudes toward technology: technology enthusiasts, pragmatists, and reluctant adopters. Similarly, another study [73] found three segments of older adults based on their Internet use patterns: non-users, instrumental users, and communicative users.

Despite the importance of designing user-friendly technology for older adults [74], the study foundthat PE variable has different levels of significance across different elder segments highlights the need to consider this demographic group’s diverse perspectives and needs. Specifically, the study found that PE is a significant predictor for independent and technological-apathetic elder segments but not for technological-eager elders. This finding suggests that technology adoption models need to consider the varying expectations and motivations of different segments of older adults to develop targeted interventions that meet their specific needs. Therefore, future research should continue to explore the nuances of technology adoption among older adults to inform better the design and implementation of technology interventions that support their digital inclusion and well-being.

Hypotheses H2, H4, and H6 of the research model were not supported. This finding implies that the variance of the intention to use SNS is not related to the variance of the EE to use SNS (H2). Likewise, the variance of FC is not associated with the variance of intention to use (H4) and SNS use (H6). Given the descriptive statistics of the items that make up these variables, we can give some possible explanations. In the case of FC, their items have a high average value with a low standard deviation, which indicates that most of the adults surveyed have the requirements to use the SNS.

On the other hand, in the case of EE, its items are not exceptionally high and have high standard deviations. This result may indicate that given the interface and functions provided by SNS, some adults find SNS easy to use, and others do not, but in no case does it disable them from using these platforms. This result may be related to the cultural characteristics of Chile; the literature explains that a high uncertainty avoidance index correlates with a non-significant relationship between EE and behavioral intention [75]. Later, some studies on the acceptance of online technologies in samples of Chilean users have shown results where this relationship is non-significant [42, 76].

## Conclusions

This research was oriented to determine segments in SNS usage by the elderly. A UTAUT2-based research model was applied to Chile’s proportional sample. The data analysis used a hybrid multigroup PLS-SEM.

The global measurement model was validated successfully, and the global structural model supported six of the nine hypotheses proposed, achieving 60.5% explanation of the use of SNS. The hybrid multigroup PLS-SEM identified three segments in the global model: independent elders, technological-apathetic elders, and technological-eager elders. The study´s results highlight that HA was the main determinant factor in the elderly accepting SNS. Still, there are statistically significant differences in the effects of the variables that explain the intention to use SNS and the use of SNS among these segments.

From a theoretical point of view, the study contributions are threefold. First, it enhances our knowledge about the variables that help segment elders’ adoption of technology, particularly SNS. In addition to the well-documented variables of gender, age, voluntariness of use, and experience, this study proposes generation and TRI profiles as advanced variables to consider when studying the heterogeneity of samples, mainly in older populations. Second, the study contributes to academic research by adding to the body of knowledge that characterizes segments in older samples. The three segments found: independent elders, technological-apathetic elders, and technological-eager elders have different behaviors when testing technology adoption models. For instance, PE, a robust variable that has been found to predict the intention to use technology across different samples and types of technology, is significant for the independent and technological-apathetic elder segments. However, it is not important for the technological-eager elder segment of the sample. Treating elders as a unique, homogeneous group may yield wrong conclusions. Third, the study uses an innovative method to segment users: the hybrid multigroup approach with Pathmox analysis offers a framework with a comprehensive data analysis process that other researchers can use.

From the practitioner´s point of view, the three segments found in the sample can interest firms and governments to treat elders differently. On the one hand, companies could create diverse marketing strategies to target elders according to the segments they belong to. For example, for the independent elder segment, the fun or pleasure derived from using SNS may not impact its intention to use it. Conversely, for the technological-eager elder segment, the HM derived from SNS use may be relevant in its intention to use it. On the other hand, governments should look closely at the differences in the elder segments to promote the use of SNS, knowing the benefits this technology can bring to elders. Moreover, the use of SNS is essential in pandemic times.

The study is not without limitations. First, using a non-random sampling method restricts the result’s generalization. Second, the study is cross-sectional; a longitudinal study would be desirable to compare different stages of SNS adoption by the elderly.

Future studies should confirm the existence of these three segments in the elderly population using an appropriate statistical technique in a larger sample. Moreover, the general analysis method proposed in this work can be used to analyze heterogeneity in acceptance models of other technologies, such as electronic health devices or learning management systems.

## Supporting information

S1 Dataset (XLSX)

S1 File Measurement instrument. (DOCX)

S2 File PLS-SEM analysis by segments. (DOCX)

## References

[1] Yap YY, Tan SH, Choon SW. Elderly’s intention to use technologies: A systematic literature review. Heliyon. 2022;8: e08765. doi: 10.1016/j.heliyon.2022.e08765 35128090 PMC8800037

[2] Jeon HG, Kim C, Lee J, Lee KC. Understanding E-Commerce Consumers’ Repeat Purchase Intention: The Role of Trust Transfer and the Moderating Effect of Neuroticism. Front Psychol. 2021;12: 1–14. doi: 10.3389/fpsyg.2021.690039 34140923 PMC8203814

[3] Blut M, Wang C. Technology readiness: a meta-analysis of conceptualizations of the construct and its impact on technology usage. J Acad Mark Sci. 2020;48: 649–669. doi: 10.1007/s11747-019-00680-8

[4] Baker S, Warburton J, Waycott J, Batchelor F, Hoang T, Dow B, et al. Combatting social isolation and increasing social participation of older adults through the use of technology: A systematic review of existing evidence. Australas J Ageing. 2018;37: 184–193. doi: 10.1111/ajag.12572 30022583

[5] Zhou J. Improving older people’s life satisfaction via social networking site use: Evidence from China. Australas J Ageing. 2018;37: 23–28. doi: 10.1111/ajag.12499 29388297

[6] Seifert A, Cotten SR, Xie B. A Double Burden of Exclusion? Digital and Social Exclusion of Older Adults in Times of COVID-19. Journals of Gerontology—Series B Psychological Sciences and Social Sciences. 2021;76: 99–103. doi: 10.1093/geronb/gbaa098 32672332 PMC7454901

[7] Sunkel G, Ullmann H. Las personas mayores de América Latina en la era digital: superación de la brecha digital. Revista de la CEPAL. 2019;2019: 243–268. doi: 10.18356/db143bd3-es

[8] OECD. Latin American Economic Outlook 2020: Digital Transformation for Building Back Better. Paris: OECD Publishing; 2020.

[9] Khoros. The 2022 Social Media Demographics Guide. 2022 [cited 20 Sep 2022]. Available: https://khoros.com/resources/social-media-demographics-guide

[10] Chopik WJ. The Benefits of Social Technology Use among Older Adults Are Mediated by Reduced Loneliness. Cyberpsychol Behav Soc Netw. 2016;19: 551–556. doi: 10.1089/cyber.2016.0151 27541746 PMC5312603

[11] Khalaila R, Vitman-Schorr A. Internet use, social networks, loneliness, and quality of life among adults aged 50 and older: mediating and moderating effects. Quality of Life Research. 2018;27: 479–489. doi: 10.1007/s11136-017-1749-4 29210015

[12] WHO. 10 Priorities: Towards a decade of healthy ageing. 2017 [cited 20 Sep 2022]. Available: https://www.who.int/news-room/feature-stories/detail/10-priorities-for-a-decade-of-action-on-healthy-ageing

[13] Rondán-Cataluña FJ, Ramírez-Correa PE, Arenas-Gaitán J, Ramírez-Santana M, Grandón EE, Alfaro-Pérez J. Social network communications in chilean older adults. Int J Environ Res Public Health. 2020;17: 6078. doi: 10.3390/ijerph17176078 32825543 PMC7503771

[14] Ramírez-Correa PE, Rondán-Cataluña FJ, Arenas-Gaitán J, Grandón EE, Alfaro-Pérez JL, Ramírez-Santana M. Segmentation of Older Adults in the Acceptance of Social Networking Sites Using Machine Learning. Front Psychol. 2021;12: 705715. doi: 10.3389/fpsyg.2021.705715 34456818 PMC8385199

[15] Casanova G, Abbondanza S, Rolandi E, Vaccaro R, Pettinato L, Colombo M, et al. New Older Users’ Attitudes Toward Social Networking Sites and Loneliness: The Case of the Oldest-Old Residents in a Small Italian City. Social Media and Society. 2021;7. doi: 10.1177/20563051211052905

[16] Villarejo-Ramos ÁF, Peral-Peral B, Arenas-Gaitán J. Latent segmentation of older adults in the use of social networks and e-banking services. Information Research. 2019;24: 841.

[17] Jones S, Fox S. Generations Online in 2009. 2009 [cited 20 Sep 2022]. Available: https://www.pewresearch.org/internet/2009/01/28/generations-online-in-2009/

[18] Peral-Peral B, Arenas-Gaitán J, Villarejo-Ramos ángel F. From digital divide to psycho-digital divide: Elders and online social networks. Comunicar. 2015;23: 57–64. doi: 10.3916/C45-2015-06

[19] Rust RT, Verhoef PC. Optimizing the marketing interventions mix in intermediate-term CRM. Marketing Science. 2005;24: 477–489. doi: 10.1287/mksc.1040.0107

[20] Sarstedt M, Ringle CM. Treating unobserved heterogeneity in PLS path modeling: A comparison of FIMIX-PLS with different data analysis strategies. J Appl Stat. 2010;37: 1299–1318. doi: 10.1080/02664760903030213

[21] Becker J-M, Rai A, Ringle CM, Völckner F. Discovering unobserved heterogeneity in structural equation models to avert validity threats. MIS Q. 2013;37: 665–694. doi: 10.25300/MISQ/2013/37.3.01

[22] Arenas-Gaitán J, Villarejo Ramos AF, Peral-Peral B. A posteriori segmentation of elderly internet users: applying PLS-POS. Marketing Intelligence and Planning. 2020;38: 340–353. doi: 10.1108/MIP-01-2019-0057

[23] Guido G, Pichierri M, Pino G, Conoci R. The Segmentation of Elderly Consumers: A Literature Review. Journal of Customer Behaviour. 2019;17: 257–278. doi: 10.1362/147539218x15445233217805

[24] Ramírez-Correa PE, Grandón EE, Arenas-Gaitán J. Assessing differences in customers’ personal disposition to e-commerce. Industrial Management and Data Systems. 2019;119: 792–820. doi: 10.1108/IMDS-07-2018-0280

[25] Parasuraman A. Technology Readiness Index (TRI): A Multiple-Item Scale to Measure Readiness to Embrace New Technologies. J Serv Res. 2000;2: 307–320. doi: 10.1177/109467050024001

[26] Rafdinal W, Senalasari W. Predicting the adoption of mobile payment applications during the COVID-19 pandemic. International Journal of Bank Marketing. 2021;39: 984–1002. doi: 10.1108/IJBM-10-2020-0532

[27] Csuka SI, Martos T, Kapornaky M, Sallay V, Lewis CA. Attitudes Toward Technologies of the near Future: The Role of Technology Readiness in a Hungarian Adult Sample. International Journal of Innovation and Technology Management. 2019;16: 1–19. doi: 10.1142/S0219877019500469

[28] Kaushik MK, Agrawal D. Influence of technology readiness in adoption of e-learning. International Journal of Educational Management. 2021;35: 483–495. doi: 10.1108/IJEM-04-2020-0216

[29] Mishra A, Maheswarappa SS, Colby CL. Technology readiness of teenagers: a consumer socialization perspective. Journal of Services Marketing. 2018;32: 592–604. doi: 10.1108/JSM-07-2017-0262

[30] Kim T, Chiu W, Chow MKF. Sport technology consumers: Segmenting users of sports wearable devices based on technology readiness. Sport, Business and Management: An International Journal. 2019;9: 134–145. doi: 10.1108/SBM-02-2018-0011

[31] Wiese M, Humbani M. Exploring technology readiness for mobile payment app users. International Review of Retail, Distribution and Consumer Research. 2019;30: 123–142. doi: 10.1080/09593969.2019.1626260

[32] Ramírez-Correa PE, Grandón EE, Rondán-Cataluña FJ. Users segmentation based on the Technological Readiness Adoption Index in emerging countries: The case of Chile. Technol Forecast Soc Change. 2020;155: 120035. doi: 10.1016/j.techfore.2020.120035

[33] Shirahada K, Ho BQ, Wilson A. Online public services usage and the elderly: Assessing determinants of technology readiness in Japan and the UK. Technol Soc. 2019;58: 101115. doi: 10.1016/j.techsoc.2019.02.001

[34] Sell A, Walden P. Segmentation of the Young Elderly Based on Technology Readiness. In: Pucihar A, Kljajić Borštnar M, Bons R, Cripps H, Sheombar A, Vidmar D, editors. Digital Support from Crisis to Progressive Change. Bled, Slovenia: AIS Electronic Library; 2021. pp. 435–450.

[35] Venkatesh V, Morris MG, Davis GB, Davis FD. User acceptance of information technology: Toward a unified view. MIS Q. 2003;27: 425–478. doi: 10.2307/30036540

[36] Venkatesh V, Thong JYL, Xu X. Consumer acceptance and use of information technology: Extending the unified theory of acceptance and use of technology. MIS Q. 2012;36: 157–178. doi: 10.2307/41410412

[37] Ramírez-Correa PE, Rondán-Cataluña FJ, Arenas-Gaitán J. An empirical analysis of mobile internet acceptance in Chile. Information Research. 2014;19: 635.

[38] Arenas-Gaitán J, Peral-Peral B, Ramón-Jerónimo MA. Elderly and internet banking: An application of UTAUT2. Journal of Internet Banking and Commerce. 2015;20: 1–23.

[39] Macedo IM. Predicting the acceptance and use of information and communication technology by older adults: An empirical examination of the revised UTAUT2. Comput Human Behav. 2017;75: 935–948. doi: 10.1016/j.chb.2017.06.013

[40] Ajzen I, Fishbein M. Understanding attitudes and predicting social behaviour. Englewood Cliffs. Englewood Cliffs, NJ: Prentice-Hall; 1980.

[41] Lorenzo Romero C, del Carmen Alarcon de Amo M, Gomez Borja MA. Adoption of social networking sites: extending the technology acceptance model integrating trust and perceived risk. Cuadernos De Economia Y Direccion De La Empresa. 2011;13: 194–205. doi: 10.1016/j.cede.2010.12.003

[42] Ramírez-Correa PE, Rondan-Cataluña FJ, Arenas-Gaitán J. Exploration of the factors that affect the adoption of social networking services by generation y in Chile. Interciencia. 2013;38: 628–633.

[43] Lankton NK, McKnight DH, Thatcher JB. The moderating effects of privacy restrictiveness and experience on trusting beliefs and habit: An empirical test of intention to continue using a social networking website. IEEE Trans Eng Manag. 2012;59: 654–665. doi: 10.1109/TEM.2011.2179048

[44] Zhou T, Li H. Understanding mobile SNS continuance usage in China from the perspectives of social influence and privacy concern. Comput Human Behav. 2014;37: 283–289. doi: 10.1016/j.chb.2014.05.008

[45] Sheikh Z, Islam T, Rana S, Hameed Z, Saeed U. Acceptance of social commerce framework in Saudi Arabia. Telematics and Informatics. 2017;34: 1693–1708. doi: 10.1016/j.tele.2017.08.003

[46] Al-Ghaith W. Applying the Technology Acceptance Model to Understand Social Networking Sites (SNS) Usage: Impact of Perceived Social Capital. International Journal of Computer Science and Information Technology. 2015;7: 105–117. doi: 10.5121/ijcsit.2015.7409

[47] Dumpit DZ, Fernandez CJ. Analysis of the use of social media in Higher Education Institutions (HEIs) using the Technology Acceptance Model. International Journal of Educational Technology in Higher Education. 2017;14: 1–16. doi: 10.1186/s41239-017-0045-2

[48] Leng GS, Lada S, Muhammad MZ, Ibrahim AAHA, Amboala T. An exploration of social networking sites (SNS) adoption in Malaysia using technology acceptance model (TAM), theory of planned behavior (TPB) and intrinsic motivation. Journal of Internet Banking and Commerce. 2011;16: 1–27.

[49] Herrero A, San Martín H, Collado J. Market orientation and SNS adoption for marketing purposes in hospitality microenterprises. Journal of Hospitality and Tourism Management. 2018;34: 30–40. doi: 10.1016/j.jhtm.2017.11.005

[50] Ismail S, Technology I, Lumpur K. International Students ‘ Acceptance on using Social Networking Site to Support Learning Activities. International Journal for Advancement of Science & Arts. 2010;1: 81–90.

[51] Kaba B, Touré B. Understanding information and communication technology behavioral intention to use: Applying the UTAUT model to social networking site adoption by young people in a least developed country. J Assoc Inf Sci Technol. 2014;65: 1662–1674. doi: 10.1002/asi.23069

[52] Moghavvemi S, Paramanathan T, Rahin NM, Sharabati M. Student’s perceptions towards using e-learning via Facebook. Behaviour and Information Technology. 2017;36: 1081–1100. doi: 10.1080/0144929X.2017.1347201

[53] Ernst C-PH, Pfeiffer J, Rothlauf F. Hedonic and utilitarian motivations of social network site usage. In: Ernst CPH, editor. Factors Driving Social Network Site Usage. Wiesbaden, Germany: Springer Gabler; 2015. pp. 11–28. doi: 10.1007/978-3-658-09918-3_2

[54] Kwon O, Wen Y. An empirical study of the factors affecting social network service use. Comput Human Behav. 2010;26: 254–263. doi: 10.1016/j.chb.2009.04.011

[55] Ramírez-Correa P, Grandón EE, Ramírez-Santana M, Órdenes LB. Explaining the use of social network sites as seen by older adults: The enjoyment component of a hedonic information system. Int J Environ Res Public Health. 2019;16: 1673. doi: 10.3390/ijerph16101673 31091670 PMC6571809

[56] Parasuraman A, Colby CL. An Updated and Streamlined Technology Readiness Index: TRI 2.0. J Serv Res. 2015;18: 59–74. doi: 10.1177/1094670514539730

[57] Lyons S, Duxbury L, Higgins C. Are gender differences in basic human values a generational phenomenon? Sex Roles. 2005;53: 763–778. doi: 10.1007/s11199-005-7740-4

[58] Morrison DF, Harman HH. Modern Factor Analysis. J Am Stat Assoc. 1961;56: 444–446. doi: 10.2307/2282293

[59] Kock N. Common method bias in PLS-SEM: A full collinearity assessment approach. International Journal of e-Collaboration. 2015;11: 1–10. doi: 10.4018/ijec.2015100101

[60] Kock N, Hadaya P. Minimum sample size estimation in PLS-SEM: The inverse square root and gamma-exponential methods. Information Systems Journal. 2018;28: 227–261. doi: 10.1111/isj.12131

[61] Hair JF, Sarstedt M, Matthews LM, Ringle CM. Identifying and treating unobserved heterogeneity with FIMIX-PLS: part I–method. European Business Review. 2016;28: 63–76. doi: 10.1108/EBR-09-2015-0094

[62] Sarstedt M. A review of recent approaches for capturing heterogeneity in partial least squares path modelling. Journal of Modelling in Management. 2008;3: 140–161. doi: 10.1108/17465660810890126

[63] Sarstedt M, Ringle CM, Hair JF. Partial Least Squares Structural Equation Modeling. In: Homburg C, Klarmann M, Vomberg A, editors. Handbook of Market Research. Cham: Springer; 2017. pp. 587–632.

[64] Wedel M, Kamakura WA. Market segmentation: Conceptual and methodological foundations. The effects of brief mindfulness intervention on acute pain experience: An examination of individual difference. New York, NY: Springer; 2000.

[65] Squillacciotti S. Prediction Oriented Classification in PLS Path Modeling. In: Vinzi E v., Chin W, Henseler J, Wang H, editors. Handbook of Partial Least Squares. Berlin, Heidelberg: Springer; 2010. pp. 219–233. doi: 10.1007/978-3-540-32827-8_10

[66] Lamberti G. Hybrid multigroup partial least squares structural equation modelling: an application to bank employee satisfaction and loyalty. Qual Quant. 2021; 1–23. doi: 10.1007/s11135-021-01096-933879929

[67] Hu LT, Bentler PM. Fit Indices in Covariance Structure Modeling: Sensitivity to Underparameterized Model Misspecification. Psychol Methods. 1998;3: 424–453. doi: 10.1037/1082-989X.3.4.424

[68] Henseler J. Bridging Design and Behavioral Research With Variance-Based Structural Equation Modeling. J Advert. 2017;46: 178–192. doi: 10.1080/00913367.2017.1281780

[69] Henseler J, Hubona G, Ray PA. Using PLS path modeling in new technology research: Updated guidelines. Industrial Management and Data Systems. 2016;116: 2–20. doi: 10.1108/IMDS-09-2015-0382

[70] Keil M, Tan BCY, Wei K-K, Saarinen T, Tuunainen V, Wassenaar A. A cross-cultural study on escalation of commitment behavior in software projects. MIS Q. 2000;24: 299–323. doi: 10.2307/3250940

[71] Sinha S, Verma A, Tiwari P. Technology: Saving and Enriching Life During COVID-19. Front Psychol. 2021;12: 647681. doi: 10.3389/fpsyg.2021.647681 33854467 PMC8040793

[72] Czaja SJ, Charness N, Fisk AD, Hertzog C, Nair SN, Rogers WA, et al. Factors predicting the use of technology: Findings from the Center for Research and Education on Aging and Technology Enhancement (CREATE). Psychol Aging. 2006;21: 333–352. doi: 10.1037/0882-7974.21.2.333 16768579 PMC1524856

[73] Xie B, Watkins I, Golbeck J, Huang M. Understanding and Changing Older Adults’ Perceptions and Learning of Social Media. Educ Gerontol. 2012;38: 282–296. doi: 10.1080/03601277.2010.544580 22639483 PMC3358790

[74] Czaja SJ, Boot WR, Charness N, Rogers WA. Designing for older adults: Principles and creative human factors approaches. 3rd ed. Boca Raton: CRC Press; 2019.

[75] Cardon PW, Marshall BA. National culture and technology acceptance: The impact of uncertainty avoidance. Issues in Information Systems. 2008;9: 103–110.

[76] Andrews L, Bianchi C. Consumer internet purchasing behavior in Chile. J Bus Res. 2013;66: 1791–1799. doi: 10.1016/j.jbusres.2013.01.012

