# Supplementary material for: Adoption of social networking sites among older adults: The role of the technology readiness and the generation to identifying segments
Source: PLoS One. 2023 Apr 18;18(4):e0284585. doi: 10.1371/journal.pone.0284585 (PMC10112803; doi:10.1371/journal.pone.0284585)
Supplement: S1 File — (DOCX) [file pone.0284585.s002.docx]

**MEASUREMENT INSTRUMENT**

Facilitating conditions

- FC1. I have the resources necessary to use social networking sites.
- FC2. I have the knowledge necessary to use social networking sites.
- FC3. The social networking sites are compatible with other technologies I use.

Effort expectancy

- EE1. Learning to use social networking sites is easy for me.
- EE2. The process of using social networking sites is clear and understandable.
- EE3. I find social networking sites easy to use.

Performance expectancy

- PU1. Social networking sites enable me to acquire more information or meet more people.
- PU2. Social networking sites would improve my efficiency in sharing information and connecting with others.
- PU3. Social networking sites are useful communication services.
- PU4. Social networking sites are a useful service for the interaction of members.

Habit

- HA1. I use social networking sites out of habit.
- HA2. Using social networking sites has become automatic for me.
- HA3. Using social networking sites is natural to me.
- HA4. I use social networking sites without thinking.
- HA5. Using social networking sites has become routine for me.

Intention to use

- USE1. I tend to use social networking sites frequently.
- USE2. I spend much time on social networking sites.

Hedonic motivation

- HM1. Using social networking sites is fun.
- HM2. Using social networking sites is enjoyable.
- HM3. Using social networking sites is very entertaining.

Social influence

- SI1. People who influence my behaviour think that I should use social networking sites.
- SI2. People who are important to me think I should use social networking sites.
- SI3. People whose opinions I value prefer me to use social networking sites.
- SI4. People I look up to expect me to use social networking sites.

Use

- U1. I use social networking sites to chat.
- U2. I use social networking sites to see photos.
- U3. I use social networking sites to see comments.
- U4. I use social networking sites to share links to other web pages.

Technology Readiness Index

- TRI1. New technologies contribute to a better quality of life.
- TRI2. Technology gives me more freedom of mobility.
- TRI3. Technology gives people more control over their daily lives.
- TRI4. Technology makes me more productive in my personal life.
- TRI5. Other people come to me for advice on new technologies.
- TRI6. In general, I am among the first in my circle of friends to acquire new technology when it appears.
- TRI7. I can usually figure out new high-tech products and services without help from others.
- TRI8. I keep up with the latest technological developments in my areas of interest.
- TRI9. When I get technical support from a high-tech product or service provider, I sometimes feel like I am being taken advantage of by someone who knows more than I do.
- TRI10. Technical support lines are not helpful because they don’t explain things in terms I understand.
- TRI11. Sometimes, I think that technology systems are not designed for use by ordinary people.
- TRI12. There is no such thing as a manual for a high-tech product or service written in plain language.
- TRI13. People are too dependent on technology to do things for them.
- TRI14. Too much technology distracts people to the point that is harmful.
- TRI15. Technology lowers the quality of relationships by reducing personal interaction.
- TRI16. I am not confidently doing business with a place that can only be reached online.

All items were measured on a 5-point Likert scale. The scale ranged from “1-never” to “5-always” for U1, U2, U3, and U4. The scale ranged from “1-strongly disagree” to “5-strongly agree” for all other items.
