# Supplementary material for: Adoption of social networking sites among older adults: The role of the technology readiness and the generation to identifying segments
Source: PLoS One. 2023 Apr 18;18(4):e0284585. doi: 10.1371/journal.pone.0284585 (PMC10112803; doi:10.1371/journal.pone.0284585)
Supplement: S2 File — (DOCX) [file pone.0284585.s003.docx]

**PLS-SEM ANALYSIS BY SEGMENTS**

**Table A1. Discriminant validity - Cross loadings (loadings and weights) for Independent elder**

| Item | FC | EE | PE | HA | IU | HM | SI | U |  |  |
| --- | --- | --- | --- | --- | --- | --- | --- | --- | --- | --- |
| FC1 | 0.772 | 0.428 | 0.494 | 0.455 | 0.433 | 0.499 | 0.250 | 0.313 |  |  |
| FC2 | 0.786 | 0.736 | 0.426 | 0.526 | 0.411 | 0.555 | 0.328 | 0.454 |  |  |
| FC3 | 0.813 | 0.309 | 0.471 | 0.396 | 0.536 | 0.465 | 0.455 | 0.453 |  |  |
| EE1 | 0.588 | 0.910 | 0.523 | 0.540 | 0.472 | 0.530 | 0.322 | 0.502 |  |  |
| EE2 | 0.573 | 0.953 | 0.561 | 0.519 | 0.462 | 0.595 | 0.296 | 0.436 |  |  |
| EE3 | 0.545 | 0.931 | 0.471 | 0.516 | 0.428 | 0.587 | 0.278 | 0.440 |  |  |
| PE1 | 0.425 | 0.547 | 0.760 | 0.473 | 0.458 | 0.569 | 0.395 | 0.512 |  |  |
| PE2 | 0.507 | 0.515 | 0.890 | 0.444 | 0.574 | 0.544 | 0.376 | 0.466 |  |  |
| PE3 | 0.391 | 0.307 | 0.800 | 0.305 | 0.452 | 0.490 | 0.432 | 0.296 |  |  |
| PE4 | 0.573 | 0.449 | 0.816 | 0.510 | 0.528 | 0.585 | 0.411 | 0.450 |  |  |
| HA1 | 0.539 | 0.546 | 0.530 | 0.882 | 0.653 | 0.634 | 0.333 | 0.643 |  |  |
| HA2 | 0.427 | 0.510 | 0.489 | 0.900 | 0.577 | 0.554 | 0.417 | 0.639 |  |  |
| HA3 | 0.657 | 0.575 | 0.441 | 0.827 | 0.619 | 0.640 | 0.412 | 0.618 |  |  |
| HA4 | 0.495 | 0.472 | 0.485 | 0.910 | 0.629 | 0.555 | 0.479 | 0.693 |  |  |
| HA5 | 0.427 | 0.395 | 0.405 | 0.898 | 0.602 | 0.482 | 0.396 | 0.683 |  |  |
| IU1 | 0.587 | 0.419 | 0.561 | 0.633 | 0.914 | 0.556 | 0.400 | 0.673 |  |  |
| IU2 | 0.480 | 0.472 | 0.567 | 0.638 | 0.907 | 0.593 | 0.465 | 0.628 |  |  |
| HM1 | 0.563 | 0.514 | 0.602 | 0.577 | 0.576 | 0.919 | 0.339 | 0.529 |  |  |
| HM2 | 0.598 | 0.632 | 0.619 | 0.603 | 0.573 | 0.903 | 0.457 | 0.491 |  |  |
| HM3 | 0.595 | 0.543 | 0.619 | 0.606 | 0.589 | 0.935 | 0.382 | 0.548 |  |  |
| SI1 | 0.333 | 0.300 | 0.396 | 0.309 | 0.363 | 0.333 | 0.829 | 0.360 |  |  |
| SI2 | 0.375 | 0.252 | 0.418 | 0.328 | 0.377 | 0.351 | 0.898 | 0.400 |  |  |
| SI3 | 0.421 | 0.225 | 0.434 | 0.455 | 0.438 | 0.406 | 0.856 | 0.426 |  |  |
| SI4 | 0.379 | 0.318 | 0.413 | 0.452 | 0.423 | 0.354 | 0.815 | 0.452 |  |  |
| U1 | 0.341 | 0.313 | 0.367 | 0.623 | 0.628 | 0.402 | 0.385 | 0.438 |  |  |
| U2 | 0.514 | 0.431 | 0.491 | 0.544 | 0.580 | 0.523 | 0.388 | 0.261 |  |  |
| U3 | 0.419 | 0.408 | 0.513 | 0.651 | 0.552 | 0.533 | 0.365 | 0.292 |  |  |
| U4 | 0.415 | 0.451 | 0.324 | 0.494 | 0.457 | 0.363 | 0.390 | 0.278 |  |  |
| **Notes**: Facilitating conditions (FC), effort expectancy (EE), performance expectancy (PE), habit (HA), intention to use (IU), hedonic motivation (HM), social influence (SI), and use (U). | | | | | | | | | | |

**Table A2. Discriminant validity - Cross loadings (loadings and weights) for Technological-apathetic elder**

| Item | FC | EE | PE | HA | IU | HM | SI | U |  |  |
| --- | --- | --- | --- | --- | --- | --- | --- | --- | --- | --- |
| FC1 | 0.766 | 0.400 | 0.229 | 0.476 | 0.219 | 0.420 | 0.219 | 0.290 |  |  |
| FC2 | 0.811 | 0.803 | 0.268 | 0.558 | 0.361 | 0.353 | 0.257 | 0.471 |  |  |
| FC3 | 0.773 | 0.386 | 0.485 | 0.364 | 0.385 | 0.440 | 0.347 | 0.307 |  |  |
| EE1 | 0.645 | 0.929 | 0.424 | 0.603 | 0.418 | 0.407 | 0.362 | 0.497 |  |  |
| EE2 | 0.690 | 0.945 | 0.363 | 0.596 | 0.298 | 0.302 | 0.245 | 0.479 |  |  |
| EE3 | 0.666 | 0.922 | 0.356 | 0.511 | 0.247 | 0.319 | 0.189 | 0.478 |  |  |
| PE1 | 0.389 | 0.347 | 0.767 | 0.452 | 0.510 | 0.424 | 0.426 | 0.464 |  |  |
| PE2 | 0.305 | 0.352 | 0.823 | 0.411 | 0.571 | 0.434 | 0.438 | 0.480 |  |  |
| PE3 | 0.339 | 0.331 | 0.798 | 0.334 | 0.550 | 0.570 | 0.464 | 0.289 |  |  |
| PE4 | 0.232 | 0.212 | 0.611 | 0.225 | 0.458 | 0.265 | 0.351 | 0.172 |  |  |
| HA1 | 0.530 | 0.618 | 0.426 | 0.875 | 0.465 | 0.521 | 0.283 | 0.610 |  |  |
| HA2 | 0.543 | 0.563 | 0.468 | 0.938 | 0.498 | 0.502 | 0.374 | 0.677 |  |  |
| HA3 | 0.644 | 0.598 | 0.409 | 0.898 | 0.552 | 0.533 | 0.350 | 0.602 |  |  |
| HA4 | 0.494 | 0.522 | 0.405 | 0.891 | 0.527 | 0.527 | 0.410 | 0.631 |  |  |
| HA5 | 0.505 | 0.506 | 0.444 | 0.917 | 0.505 | 0.469 | 0.403 | 0.716 |  |  |
| IU1 | 0.398 | 0.333 | 0.660 | 0.539 | 0.920 | 0.564 | 0.505 | 0.405 |  |  |
| IU2 | 0.370 | 0.324 | 0.601 | 0.482 | 0.898 | 0.490 | 0.556 | 0.301 |  |  |
| HM1 | 0.574 | 0.476 | 0.611 | 0.550 | 0.559 | 0.904 | 0.424 | 0.451 |  |  |
| HM2 | 0.419 | 0.286 | 0.438 | 0.522 | 0.506 | 0.908 | 0.390 | 0.339 |  |  |
| HM3 | 0.387 | 0.261 | 0.501 | 0.468 | 0.525 | 0.927 | 0.402 | 0.318 |  |  |
| SI1 | 0.288 | 0.212 | 0.469 | 0.334 | 0.450 | 0.393 | 0.874 | 0.363 |  |  |
| SI2 | 0.179 | 0.132 | 0.429 | 0.230 | 0.373 | 0.306 | 0.848 | 0.302 |  |  |
| SI3 | 0.205 | 0.095 | 0.432 | 0.295 | 0.375 | 0.300 | 0.784 | 0.279 |  |  |
| SI4 | 0.398 | 0.429 | 0.463 | 0.399 | 0.602 | 0.403 | 0.746 | 0.353 |  |  |
| U1 | 0.148 | 0.421 | 0.354 | 0.429 | 0.320 | 0.348 | 0.288 | 0.315 |  |  |
| U2 | 0.264 | 0.271 | 0.313 | 0.421 | 0.379 | 0.355 | 0.272 | 0.206 |  |  |
| U3 | 0.471 | 0.450 | 0.412 | 0.601 | 0.298 | 0.303 | 0.363 | 0.543 |  |  |
| U4 | 0.324 | 0.255 | 0.211 | 0.484 | 0.146 | 0.170 | 0.184 | 0.350 |  |  |
| **Notes**: Facilitating conditions (FC), effort expectancy (EE), performance expectancy (PE), habit (HA), intention to use (IU), hedonic motivation (HM), social influence (SI), and use (U). | | | | | | | | | | |

**Table A3. Discriminant validity - Cross loadings (loadings and weights) for Technological-eager elder**

| Item | FC | EE | PE | HA | IU | HM | SI | U |  |  |
| --- | --- | --- | --- | --- | --- | --- | --- | --- | --- | --- |
| FC1 | 0.720 | 0.524 | 0.357 | 0.385 | 0.356 | 0.426 | 0.332 | 0.289 |  |  |
| FC2 | 0.762 | 0.545 | 0.297 | 0.313 | 0.284 | 0.244 | 0.161 | 0.308 |  |  |
| FC3 | 0.822 | 0.366 | 0.516 | 0.468 | 0.500 | 0.447 | 0.508 | 0.498 |  |  |
| EE1 | 0.622 | 0.942 | 0.382 | 0.458 | 0.326 | 0.414 | 0.211 | 0.408 |  |  |
| EE2 | 0.490 | 0.930 | 0.309 | 0.375 | 0.198 | 0.285 | 0.125 | 0.294 |  |  |
| EE3 | 0.470 | 0.874 | 0.253 | 0.319 | 0.205 | 0.259 | 0.183 | 0.236 |  |  |
| PE1 | 0.302 | 0.257 | 0.728 | 0.452 | 0.337 | 0.448 | 0.384 | 0.478 |  |  |
| PE2 | 0.496 | 0.314 | 0.894 | 0.561 | 0.613 | 0.670 | 0.585 | 0.684 |  |  |
| PE3 | 0.457 | 0.232 | 0.896 | 0.411 | 0.503 | 0.531 | 0.576 | 0.455 |  |  |
| PE4 | 0.518 | 0.392 | 0.901 | 0.439 | 0.621 | 0.603 | 0.639 | 0.507 |  |  |
| HA1 | 0.478 | 0.431 | 0.577 | 0.911 | 0.531 | 0.705 | 0.409 | 0.744 |  |  |
| HA2 | 0.463 | 0.360 | 0.516 | 0.949 | 0.535 | 0.628 | 0.380 | 0.743 |  |  |
| HA3 | 0.480 | 0.444 | 0.327 | 0.765 | 0.456 | 0.605 | 0.323 | 0.519 |  |  |
| HA4 | 0.379 | 0.289 | 0.438 | 0.877 | 0.475 | 0.556 | 0.415 | 0.646 |  |  |
| HA5 | 0.491 | 0.387 | 0.494 | 0.891 | 0.531 | 0.600 | 0.457 | 0.676 |  |  |
| IU1 | 0.538 | 0.292 | 0.582 | 0.508 | 0.909 | 0.551 | 0.668 | 0.547 |  |  |
| IU2 | 0.393 | 0.213 | 0.550 | 0.533 | 0.901 | 0.527 | 0.575 | 0.576 |  |  |
| HM1 | 0.467 | 0.330 | 0.670 | 0.595 | 0.575 | 0.930 | 0.461 | 0.602 |  |  |
| HM2 | 0.454 | 0.365 | 0.598 | 0.651 | 0.500 | 0.932 | 0.407 | 0.595 |  |  |
| HM3 | 0.467 | 0.325 | 0.589 | 0.711 | 0.579 | 0.925 | 0.425 | 0.666 |  |  |
| SI1 | 0.457 | 0.188 | 0.532 | 0.395 | 0.602 | 0.372 | 0.908 | 0.376 |  |  |
| SI2 | 0.424 | 0.173 | 0.544 | 0.413 | 0.555 | 0.391 | 0.901 | 0.400 |  |  |
| SI3 | 0.412 | 0.142 | 0.591 | 0.408 | 0.634 | 0.470 | 0.919 | 0.386 |  |  |
| SI4 | 0.333 | 0.176 | 0.586 | 0.346 | 0.584 | 0.372 | 0.729 | 0.410 |  |  |
| U1 | 0.425 | 0.359 | 0.485 | 0.640 | 0.472 | 0.522 | 0.341 | 0.451 |  |  |
| U2 | 0.362 | 0.289 | 0.444 | 0.532 | 0.533 | 0.505 | 0.305 | 0.323 |  |  |
| U3 | 0.419 | 0.226 | 0.570 | 0.651 | 0.512 | 0.589 | 0.438 | 0.475 |  |  |
| U4 | 0.404 | 0.256 | 0.472 | 0.407 | 0.381 | 0.414 | 0.332 | -0.020 |  |  |
| **Notes**: Facilitating conditions (FC), effort expectancy (EE), performance expectancy (PE), habit (HA), intention to use (IU), hedonic motivation (HM), social influence (SI), and use (U). | | | | | | | | | | |

**Table A4. Construct coefficients by segments.**

| Segment | Coeff. | FC | EE | PE | HA | IU | HM | SI |  |
| --- | --- | --- | --- | --- | --- | --- | --- | --- | --- |
| Independent elder | AVE | 0.625 | 0.867 | 0.669 | 0.781 | 0.829 | 0.844 | 0.723 |  |
|  | CA | 0.703 | 0.923 | 0.834 | 0.930 | 0.794 | 0.908 | 0.872 |  |
|  | CR | 0.833 | 0.951 | 0.890 | 0.947 | 0.906 | 0.942 | 0.912 |  |
|  | rho_A | 0.711 | 0.925 | 0.846 | 0.930 | 0.795 | 0.908 | 0.875 |  |
| Technological-apathetic elder | AVE | 0.614 | 0.869 | 0.569 | 0.817 | 0.826 | 0.833 | 0.663 |  |
|  | CA | 0.695 | 0.928 | 0.742 | 0.944 | 0.790 | 0.900 | 0.835 |  |
|  | CR | 0.827 | 0.952 | 0.839 | 0.957 | 0.905 | 0.938 | 0.887 |  |
|  | rho_A | 0.712 | 0.996 | 0.753 | 0.946 | 0.797 | 0.902 | 0.852 |  |
| Technological-eager elder | AVE | 0.592 | 0.839 | 0.736 | 0.776 | 0.819 | 0.863 | 0.753 |  |
|  | CA | 0.671 | 0.906 | 0.880 | 0.926 | 0.779 | 0.921 | 0.887 |  |
|  | CR | 0.813 | 0.940 | 0.917 | 0.945 | 0.900 | 0.950 | 0.924 |  |
|  | rho_A | 0.709 | 0.996 | 0.917 | 0.936 | 0.780 | 0.926 | 0.890 |  |
| **Notes:** Cronbach's alpha (CA), composite reliability (CR), average variance extracted (AVE), facilitating conditions (FC), effort expectancy (EE), performance expectancy (PE), habit (HA), intention to use (IU), hedonic motivation (HM), social influence (SI), and use (U). | | | | | | | | | |

**Table A5. Construct discriminant validity - Fornell-Larcker criterion by segments.**

| Construct | FC | EE | PE | HA | IU | HM | SI | U |
| --- | --- | --- | --- | --- | --- | --- | --- | --- |
| *Independent elder* | | | | | | | | |
| FC | 0.791 |  |  |  |  |  |  |  |
| EE | 0.611 | 0.931 |  |  |  |  |  |  |
| PE | 0.584 | 0.558 | 0.818 |  |  |  |  |  |
| HA | 0.576 | 0.564 | 0.532 | 0.884 |  |  |  |  |
| IU | 0.587 | 0.489 | 0.619 | 0.698 | 0.910 |  |  |  |
| HM | 0.637 | 0.612 | 0.668 | 0.648 | 0.631 | 0.919 |  |  |
| SI | 0.447 | 0.322 | 0.490 | 0.461 | 0.474 | 0.427 | 0.850 |  |
| U | 0.521 | 0.494 | 0.529 | 0.742 | 0.715 | 0.569 | 0.485 | Composite |
| *Technological-apathetic elder* | | | | | | | | |
| FC | 0.784 |  |  |  |  |  |  |  |
| EE | 0.712 | 0.932 |  |  |  |  |  |  |
| PE | 0.421 | 0.416 | 0.754 |  |  |  |  |  |
| HA | 0.600 | 0.619 | 0.476 | 0.904 |  |  |  |  |
| IU | 0.423 | 0.362 | 0.695 | 0.563 | 0.909 |  |  |  |
| HM | 0.507 | 0.377 | 0.569 | 0.563 | 0.582 | 0.913 |  |  |
| SI | 0.353 | 0.302 | 0.559 | 0.404 | 0.582 | 0.445 | 0.814 |  |
| U | 0.470 | 0.522 | 0.474 | 0.717 | 0.392 | 0.407 | 0.408 | Composite |
| *Technological-eager elder* | | | | | | | | |
| FC | 0.769 |  |  |  |  |  |  |  |
| EE | 0.591 | 0.916 |  |  |  |  |  |  |
| PE | 0.531 | 0.354 | 0.858 |  |  |  |  |  |
| HA | 0.519 | 0.431 | 0.541 | 0.881 |  |  |  |  |
| IU | 0.517 | 0.280 | 0.626 | 0.575 | 0.905 |  |  |  |
| HM | 0.498 | 0.365 | 0.668 | 0.703 | 0.596 | 0.929 |  |  |
| SI | 0.470 | 0.195 | 0.652 | 0.452 | 0.688 | 0.465 | 0.868 |  |
| U | 0.499 | 0.357 | 0.623 | 0.762 | 0.620 | 0.670 | 0.454 | Composite |
| **Notes:** Facilitating conditions (FC), effort expectancy (EE), performance expectancy (PE), habit (HA), intention to use (IU), hedonic motivation (HM), social influence (SI), and use (U). | | | | | | | | |

**Table A6. Construct discriminant validity - Heterotrait-Monotrait Ratio by segments.**

| Construct | FC | EE | PE | HA | IU | HM |
| --- | --- | --- | --- | --- | --- | --- |
| *Independent elder* | | | | | | |
| EE | 0.768 |  |  |  |  |  |
| PE | 0.760 | 0.632 |  |  |  |  |
| HA | 0.718 | 0.610 | 0.602 |  |  |  |
| IU | 0.777 | 0.571 | 0.756 | 0.812 |  |  |
| HM | 0.801 | 0.670 | 0.769 | 0.706 | 0.743 |  |
| SI | 0.552 | 0.358 | 0.577 | 0.505 | 0.567 | 0.478 |
| *Technological-apathetic elder* | | | | | | |
| EE | 0.838 |  |  |  |  |  |
| PE | 0.581 | 0.488 |  |  |  |  |
| HA | 0.732 | 0.654 | 0.566 |  |  |  |
| IU | 0.549 | 0.401 | 0.900 | 0.651 |  |  |
| HM | 0.645 | 0.397 | 0.686 | 0.611 | 0.686 |  |
| SI | 0.430 | 0.285 | 0.698 | 0.432 | 0.680 | 0.494 |
| *Technological-eager elder* | | | | | | |
| EE | 0.770 |  |  |  |  |  |
| PE | 0.636 | 0.377 |  |  |  |  |
| HA | 0.639 | 0.461 | 0.594 |  |  |  |
| IU | 0.674 | 0.314 | 0.730 | 0.677 |  |  |
| HM | 0.609 | 0.382 | 0.728 | 0.762 | 0.700 |  |
| SI | 0.557 | 0.211 | 0.721 | 0.499 | 0.825 | 0.512 |
| **Notes:** Facilitating conditions (FC), effort expectancy (EE), performance expectancy (PE), habit (HA), intention to use (IU), hedonic motivation (HM), and social influence (SI). | | | | | | |
